# Alternate Partial Root-Zone Drip Nitrogen Fertigation Reduces Residual Nitrate Loss While Improving the Water Use but Not Nitrogen Use Efficiency

**DOI:** 10.3389/fpls.2021.722459

**Published:** 2021-10-13

**Authors:** Rui Liu, Peng-Fei Zhu, Yao-Sheng Wang, Zhen Chen, Ji-Rong Zhu, Liang-Zuo Shu, Wen-Ju Zhang

**Affiliations:** ^1^Zhejiang Provincial Key Laboratory of Plant Evolutionary and Conservation, School of Life Science, Taizhou University, Taizhou, China; ^2^Anhui Key Laboratory of Resource and Plant Biology, School of Life Sciences, Huaibei Normal University, Huaibei, China; ^3^Laboratory of Dryland Agriculture, Institute of Environment and Sustainable Development in Agriculture, Chinese Academy of Agricultural Sciences, Beijing, China; ^4^National Engineering Laboratory for Improving Quality of Arable Land, Institute of Agricultural Resources and Regional Planning, Chinese Academy of Agricultural Sciences, Beijing, China

**Keywords:** root growth, fruit yield, irrigation water use efficiency, nitrogen use efficiency, ^15^N

## Abstract

The efficient utilization of irrigation water and nitrogen is of great importance for sustainable agricultural production. Alternate partial root-zone drip irrigation (APRD) is an innovative water-saving drip irrigation technology. However, the coupling effects of water and nitrogen (N) supply under APRD on crop growth, water and N use efficiency, as well as the utilization and fate of residual nitrates accumulated in the soil profile are not clear. A simulated soil column experiment where 30–40 cm soil layer was ^15^NO_3_-labeled as residual nitrate was conducted to investigate the coupling effects of different water [sufficient irrigation (W_1_), two-thirds of the W_1_(W_2_)] and N [high level (N_1_), 50% of N_1_ (N_2_)] supplies under different irrigation modes [conventional irrigation (C), APRD (A)] on tomato growth, irrigation water (IWUE) and N use efficiencies (NUE), and the fate of residual N. The results showed that, compared with CW_1_N_1_, AW_1_N_1_ promoted root growth and nitrogen absorption, and increased tomato yield, while the N absorption and yield did not vary significantly in AW_2_N_1_. The N absorption in AW_2_N_2_ decreased by 16.1%, while the tomato yield decreased by only 8.8% compared with CW_1_N_1_. The highest IWUE appeared in AW_2_N_1_, whereas the highest NUE was observed in AW_2_N_2_, with no significant difference in NUE between AW_2_N_1_ and CW_1_N_1_ at the same N supply level. The ^15^N accumulation peak layer was almost the same as the originally labeled layer under APRD, whereas it moved 10–20 cm downwards under CW_1_N_1_. The amount of ^15^N accumulated in the 0-40 cm layer increased with the decreasing irrigation water and nitrogen supply, with an increase of 82.9–141.1% in APRD compared with that in CW_1_N_1_. The utilization of the ^15^N labeled soil profile by the tomato plants increased by 9–20.5%, whereas the loss rate of ^15^N from the plant-soil column system decreased by 21.3–50.1% in APRD compared with the CW_1_N_1_ treatment. Thus, APRD has great potential in saving irrigation water, facilitating water use while reducing the loss of residual nitrate accumulated in the soil profile, but has no significant effect on the NUE absorbed.

## Introduction

Water shortage is a great concern that is jeopardizing sustainable development globally, including in China. Water crisis coexists with the low efficiency of irrigation water in agricultural production, which consumes ~70% of the total freshwater (Mancosu et al., 2015; Kang et al., 2017). Developing water-saving irrigation technologies is an essential and urgent requirement to support the high food demand for the increasing world population. Deficit irrigation and alternate partial root-zone irrigation (APRI) are two water-saving irrigation strategies currently investigated (Kang and Zhang, 2004; Dodd, 2009; Sezen et al., 2019). In deficit irrigation, the amount of water supplied to the whole root zone is less than that of plant evapotranspiration, inducing moderate water stress in the plant which has marginal effects on yield formation (Dodd, 2009). In APRI, irrigation is supplied only to half of the root system, leaving the other half dry till the next irrigation occurs. The repeated alternation of wetting/drying in the two root zones in APRI induces an abscisic acid (ABA)-based root-to-shoot chemical signaling, hydraulic signals, and an increased xylem sap pH to regulate the stomatal opening thereby increasing water use efficiency (WUE) (Kang and Zhang, 2004; Hu et al., 2011; Pérez-Pérez et al., 2018). For many plant species including cotton, corn, tomato, potato, cucumber, grape, and apple, APRI has been demonstrated to be an efficient water-saving irrigation technology that outperforms deficit irrigation by maintaining the yield and improving the WUE substantially (Kang and Zhang, 2004; Shahnazari et al., 2007; Dodd, 2009; Yactayo et al., 2013; Jovanovic and Stikic, 2018; Sarker et al., 2019). The main form in APRI application is the alternate furrow irrigation or alternate watering to different sides of the plants (Yactayo et al., 2013; Zhang et al., 2014; Sarker et al., 2019, 2020; Khalili et al., 2020). However, it is a time-consuming and laborious process to manipulate the furrows or to manually switch the irrigation sides, which limits its use in practice. With the popularization of drip fertilization technology, an emerging new kind of APRI, named alternate partial root-zone drip irrigation (APRD) is formed by combining drip irrigation with APRI (Du et al., 2008a,b; Topak et al., 2016; Sezen et al., 2019). This form of APRI is not only easy to implement but also potentially has the advantages of both APRI and drip irrigation in improving WUE and yield (Topak et al., 2016; Sezen et al., 2019; Liu et al., 2020).

Nitrogen is an important macro-nutrient for crop growth and yield. However, the excessive application of nitrogen (N) fertilizer not only reduces fertilizer use efficiency but also produces many environmental problems, adversely impacting the quality of vegetables and fruit trees (Zhu and Chen, 2002; Gong et al., 2011). In addition, excessive fertilization leads to the accumulation of high amounts of residual nitrate in the soil profile of farmlands, which could leach into the groundwater, causing environmental problems (Zhu and Chen, 2002; Gathumbi et al., 2003). Therefore, increasing the N use efficiency (NUE) and reducing the soil residual nitrate accumulation and its leaching into the groundwater is an important issue to be resolved (Gathumbi et al., 2003).

Alternate partial root-zone irrigation can facilitate the accumulation of nitrate in the topsoil, promote the absorption of N by plants, and reduce the potential risk of nitrate leaching (Tafteh and Sepaskhah, 2012; Wang et al., 2014, 2020; Hou et al., 2017). However, whether the NUE in plants has improved under APRI is unclear. Although APRD is a promising new technology, the coupling effects of different water and N supplies on the movement and utilization of the residual nitrate accumulated in the soil profile under APRD are not known. Furthermore, the residual nitrate accumulated 30–40 cm under APRI also received little attention. Root growth and distribution decreased sharply beneath 20 cm, while it accumulated higher amounts of residual nitrate in the 20–40 cm layer (Zhang et al., 2014; Liu et al., 2020). Therefore, in the present study, a soil column experiment was conducted with the ^15^N-labeled K^15^NO_3_ as the residual nitrate in the 30–40 cm soil layer, to investigate the effects of different water and nitrogen supply on the growth, WUE, NUE, and the fate of residual nitrate accumulated in the soil profile of tomato plants, a common greenhouse vegetable, under APRD. The outcome of this study would be of great significance to guide efficient utilization of water and N resources and reduce environmental risk by sustainable agricultural production.

## Materials and Methods

### Experimental Site

The experimental site is located in a steel-framed vegetable greenhouse in Xuji Village (116°46′E, 33°58′N), Duji District, Huaibei City, Anhui Province, China. It belongs to a typical temperate humid climate, with an average annual relative humidity of 71%, an annual average frost-free period of 202 days, and sunshine hours of 2315.8 h. The experiment was initiated on February 28 and finished on June 25, 2017. The experimental soil was sandy loam (Shu et al., 2020). The pH of the soil was 7.3, with 26.2% (gravimetric) or 0.341 cm^−3^ field water capacity, 1.6 g kg^−1^ total N, 37.4 mg kg^−1^ nitrate N, 0.6 g kg^−1^ total phosphorus (P), and 1.3 g cm^−3^ soil bulk density.

### Experimental Treatments

**Figure 1:**
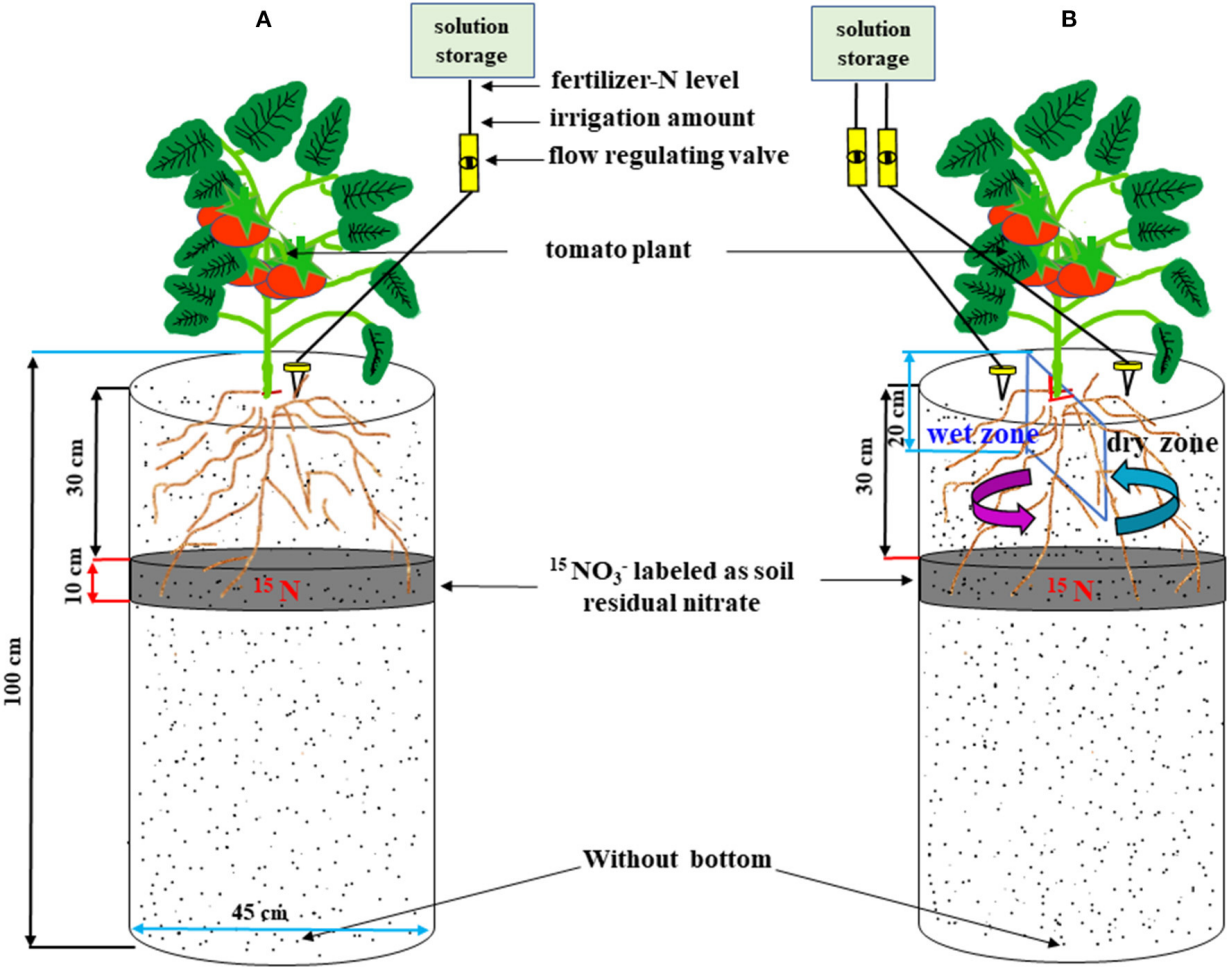
The schematic view of the soil column design for conventional drip irrigation **(A)** and alternate partial root-zone drip irrigation **(B)**.

The experiment was performed using soil columns which were made using cylindrical aluminum drums (Figure 1) as described in our previous studies (Wang et al., 2019, 2020; Liu et al., 2020). A ditch was dug in the center of the greenhouse and the soil from the layers 0–20, 20–40, 40–60, and 60–100 cm were separated. Eighteen homemade bottomless cylindrical aluminum drums with a height of 105 cm and a diameter of 45 cm were put vertically to a depth of 100 cm along the ditch, 15 cm apart from each other. Then the sieved dry soil (passed through a 2 mm sieve) from the original layers was backfilled to the drums and watered to its 90% field capacity layer by layer. While filling the soil columns, the ditch area outside the drums was also filled. In the 30–40 cm layer of each column, the soil was mixed and labeled with 11.9 g K^15^NO_3_ (the abundance of ^15^N was 20.3%, provided by the Shanghai Research Institute of Chemical Industry). All the P and potassium fertilizers were supplied as basal fertilizers and were mixed with the top 0–20 cm soil layer as KH_2_PO_4_ and K_2_SO_4_ at the rate of 200 mg P_2_O_5_ kg^−1^ and 300 mg K_2_O kg^−1^, respectively. Nitrogen fertilizer was supplied by fertigation while planting the tomatoes. To avoid the surface flow of irrigated water between the root compartments as indicated previously in the pot APRI experiments (Li et al., 2010; Wang et al., 2010), the top 0-20 cm soil in the APRD column was separated into two equal compartments by inserting a plastic film vertically in the center (Hou et al., 2017; Liu et al., 2020). A gap of 5 × 5 cm size was cut in the center of the plastic film to allow the transplanting of tomato seedlings.

The method for supplying water and N was the same as in our previous study (Liu et al., 2020). Briefly, a 5 L plastic bucket was suspended at a height of 1.8 m above the soil column (Figure 1). The water and N fertilizers were delivered from the bucket to the surface of the soil column through a medical infusion tube with needles (specification 16G, aperture 1.3 mm). On February 28, 2017, 50-day-old tomato (*Lycopersicon esculentum* Mill., cv. Zhongyan No. 958) seedlings of uniform size were transplanted in the center of the columns, with one seedling for each column. The surface layer of the column was covered with a plastic film after transplanting to reduce water evaporation. The plants were watered with conventional drip irrigation during the plant re-establishment stage. The water and N fertilizer treatments were initiated 18 days after transplantation (March 18). The four treatments included CW_1_N_1_, AW_1_N_1_, AW_2_N_1_, and AW_2_N_2_, where C represented conventional drip irrigation, A represented APRD, W_1_ and W_2_ represented sufficient and deficient irrigation, and N_1_ and N_2_ represented high N and low N application rate, respectively. Each treatment was replicated four times.

In the CW_1_N_1_ treatment, the soil in 0–20 cm (seedling stage) or 0–30 cm (after flowering stage) was irrigated to 90% of the field capacity whenever the soil water content dropped to 65% of the field capacity. The irrigation amount of W_1_ (CW_1_N_1_) was calculated according to the method described by Liu et al. (2020). Irrigation was applied to all treatments when CW_1_N_1_ was irrigated. For the deficient irrigation treatment, two-thirds of the W_1_ (W_2_) irrigation amount was applied. For the N application amount, N_1_ was calculated as supplying N at 240 mg kg^−1^ to the 0–20 cm soil layer in the column, and N_2_ received 50% of the N_1_ level. The N fertilizer was supplied as urea at 8.30 g urea per column for the N_1_ level during the experiment. In the CW_1_N_1_ treatment, fertigation was supplied to a location 5-cm away from the plants through needles (Figure 1A). In the APRD treatment (AW_1_N_1_, AW_2_N_1_, and AW_2_N_2_), fertigation was supplied alternately to only one compartment in the center of each irrigation event, letting the other soil compartment dry. At the next irrigation, the fertigation was shifted to the previously dry compartment, letting the previously irrigated compartment dry (Figure 1B). The N fertigation interval was at 4–10 days depending on the soil water content in the soil columns. There were a total of fourteen drip fertigation events in the present study, with 0.593 or 0.296 g urea at each fertigation for the N_1_ or N_2_ treatment, respectively, forming seven alternating wetting–drying fertigation cycles in each root compartment of the AW_1_N_1_-, AW_2_N_1_-, and AW_2_N_2_-treated soil columns.

To monitor the changes in the soil water content and to determine when to start the irrigation, two soil columns without the ^15^N labeling were set as the reference and were managed as the CW_1_N_1_ treatment. A time-domain reflection meter (TDR) instrument (TRIME-PICO-IPH-TDR, IMKO, Germany) was buried in the reference column at a depth of 0–100 cm. In addition, a portable TDR soil moisture meter was also used for examining the soil water content in the 0–20 cm soil layer. When the soil moisture content declined to 65% of the field capacity, irrigation was applied to all columns. The amount of water supplied was 8.5 L for each column before different drip fertigation treatments were initiated. After that, 42.7 L or 28.2 L of irrigation water was supplied to the treatments as W_1_ and W_2_, respectively. Therefore, the W_2_ treatment saved 28.3% of irrigation water in the whole plant growth period compared with that of W_1_. The plants had five ears of fruit per plant and there were three fruits per ear. The tomato plants were finally harvested on June 25, 2017.

### Measurements and Methods

On May 5, after removing the apical buds, the plant height and stem diameter were measured. The plant height was measured with a tape. The diameter of the stem (3 cm above the ground) was measured with a 0.01 mm precision digital vernier caliper (Shanghai Meinaite Industrial Inc., Shanghai, China).

The fallen leaves on the ground were collected during plant growth, and the fruits were harvested successively to maturity. The plants were harvested on June 25, 2017, and were divided into leaves, stems, and fruits. All the fresh samples were weighed and then oven-dried at 105°C for 30 min immediately after sampling and thereafter dried at 70°C to constant mass. The biomass of the leaves included the fallen leaves. The fruit yield was the cumulative value of fruits collected in different batches.

After harvest, the soil outside the soil columns was dug out, then the aluminum drum was exposed and cut longitudinally. About 20 cm per layer of soil was taken out from the drums horizontally. The roots were collected carefully using tweezers and then cleaned with distilled water. After root samples in each layer were collected, the soil was mixed thoroughly in a basin and soil samples were taken. A subsample was used to determine the soil water content immediately. The remaining soil samples were air-dried and then used for determining the total N content and the soil ^15^N.

The roots were scanned by a root scanner (Epson Perfection V700 Photo, Epson, Japan), and the root length, diameter, and surface area were analyzed with a WinRhizoPro Vision 5.0 (Regent Instruments, Inc., Quebec, Canada). After scanning, the roots in each layer were dried in the oven to constant mass.

Both the plant and soil samples were ground and passed through a 0.25-mm sieve. The concentrations of the total N and ^15^N were analyzed using mass spectrometry (isoprime100, Elementar Analysensysteme GmbH, Germany) coupled with an elemental analyzer (Vario pyro cube, Analysensysteme GmbH, Germany). The total N absorption was calculated by N concentration and the dry mass of each organ.

Irrigation water use efficiency (kg·m^−3^) = fresh mass of tomato fruit/total irrigation water used. NUE (g·g^−1^) = dry mass of tomato fruit/N accumulated in the plant. ^15^N absorption (mg) = (atom% ^15^N excess of total N in plant) × (total N in the plant). The utilization of ^15^N by the plants (%) = (atom% ^15^N excess of total N in the plant) × (total N in the plant)/(amount of ^15^N labeled). Soil ^15^N accumulation (mg) = (atom% ^15^N excess of soil total N) × (total N per soil layer).

### Statistical Analysis

The experimental data were analyzed using a one-way ANOVA with the SPSS software (IBM, Armonk, NY, USA) and average comparisons were made using Duncan's multiple range test at *P* ≤ 0.05. The data were expressed as mean ± standard error.

## Results

### Effects of Irrigation and Nitrogen Supply on Plant Growth

**Figure 2:**
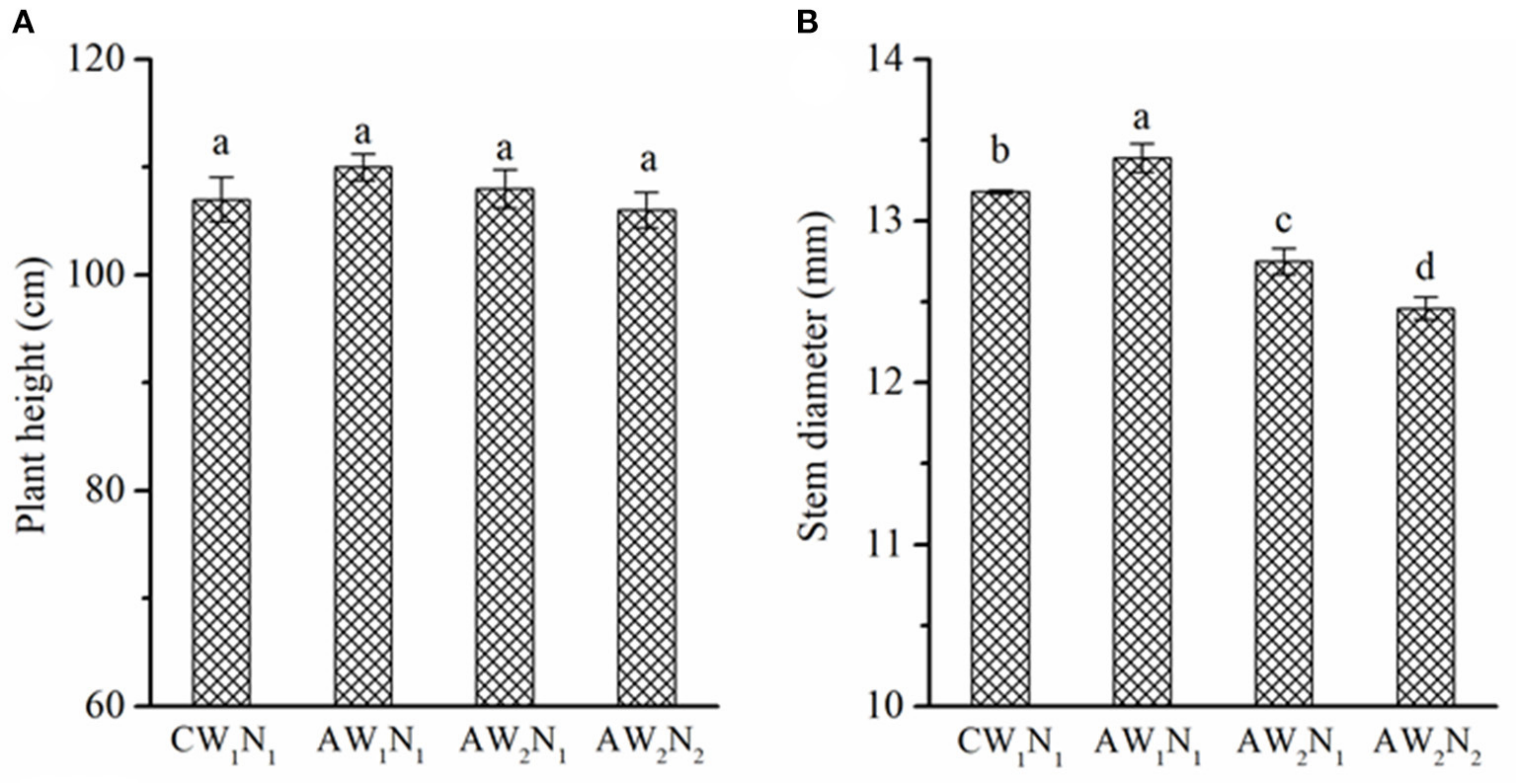
The effects of irrigation and nitrogen (N) supply on the plant height (**A**, cm) and stem diameter (**B**, mm) of tomato plants under alternate partial root-zone drip irrigation. Values are the means ± SE. Different lowercase letters in the columns denote significant differences among the treatments (*P* ≤ 0.05).

The irrigation methods and the amount of irrigation water or N supplied did not affect the height of the tomato plants (Figure 2A), but significantly affected the stem diameter (Figure 2B). Compared with the CW_1_N_1_ treatment, the stem diameter under the AW_1_N_1_ treatment increased by 1.6%, whereas it decreased by 3.3 and 5.5% under the AW_2_N_1_ and AW_2_N_2_ treatment, respectively. Under APRD, a reduction in one-third of the irrigation water decreased the stem diameter by 4.8% in the AW_2_N_1_ treatment compared with AW_1_N_1_.

Compared with CW_1_N_1_, the leaf and total biomass and fruit mass in the AW_1_N_1_ treatment increased significantly except for the stem dry mass (Table 1). Under APRD, the stem, leaf, and total dry biomass and fruit mass in the AW_2_N_1_ treatment were significantly lower than those of AW_1_N_1_. Compared with the CW_1_N_1_ treatment, the total biomass in the AW_2_N_1_ treatment decreased by 5.8%, while the fruit mass did not decrease significantly. Under the APRD with W_2_ and a reduction in N fertilizer by 50%, there were no significant effects on the fruit yield and leaf biomass but decreased the stem biomass and total biomass by 8.4% on average (AW_2_N_2_ vs. AW_2_N_1_). Compared with CW_1_N_1_, the fruit yield in AW_2_N_2_ decreased by only 8.8%, even though the irrigation amount and nitrogen application rate were both decreased substantially in the AW_2_N_2_.

**Table 1:** Effects of irrigation and nitrogen (N) supply on tomato biomass (g plant^−1^) and yield (g plant^−1^) under alternate partial root-zone drip N fertigation.

**Treatments**	**Stem dry mass**	**Leaf dry mass**	**Fruit dry mass**	**Fruit fresh mass**	Total biomass^*^
CW_1_N_1_	91.0 ± 2.8ab	80.7 ± 1.0b	220.9 ± 3.9b	4270 ± 29b	403.2 ± 5.4b
AW_1_N_1_	95.3 ± 0.8a	88.0 ± 0.7a	259.5 ± 2.5a	4573 ± 24a	453.9 ± 3.2a
AW_2_N_1_	87.6 ± 0.3b	72.7 ± 2.7c	208.9 ± 7.8bc	4117 ± 64b	379.7 ± 7.1c
AW_2_N_2_	77.8 ± 1.8c	68.0 ± 1.6c	202.6 ± 2.7c	3874 ± 38c	358.0 ± 2.5d

* *The total biomass was the sum of the dry mass of root, stem, leaf, and fruit. Values are the means ± SE. The different lowercase letters in the column denote significant differences among the treatments (P ≤ 0.05)*.

### Effects of Irrigation and Nitrogen Supply on Root Growth and Distribution

Compared with CW_1_N_1_, AW_1_N_1_ increased the root length and surface area by 6.8 and 8.7%, respectively, while there was no significant difference in the root dry mass (Table 2). There was no significant difference in the root dry mass, length, and surface area between the AW_2_N_1_ and CW_1_N_1_ treatments. However, the root length and root surface area were lower in AW_2_N_1_ than those of AW_1_N_1_. The AW_2_N_2_ treatment had no significant influence on the root length but decreased the root dry mass by 9.4% and the root surface area by 12.5% compared with the CW_1_N_1_ treatment.

**Table 2:** The effects of irrigation and N supply on the mass, length, and surface area of tomato roots under alternate partial root-zone drip N fertigation.

**Treatments**	**Dry mass (g plant^**–1**^)**	**Length (m plant^**–1**^)**	**Surface area (cm^**2**^ plant^**–1**^)**
CW_1_N_1_	10.60 ± 0.12a	226.3 ± 0.6b	9009 ± 153b
AW_1_N_1_	11.11 ± 0.09a	241.8 ± 4.3a	9792 ± 150a
AW_2_N_1_	10.58 ± 0.13a	230.6 ± 3.8b	9243 ± 118b
AW_2_N_2_	9.60 ± 0.39b	221.6 ± 1.2b	7882 ± 160c

*The values are the means ± SE. The different lowercase letters in the columns denote significant differences among the treatments (P ≤ 0.05)*.

**Figure 3:**
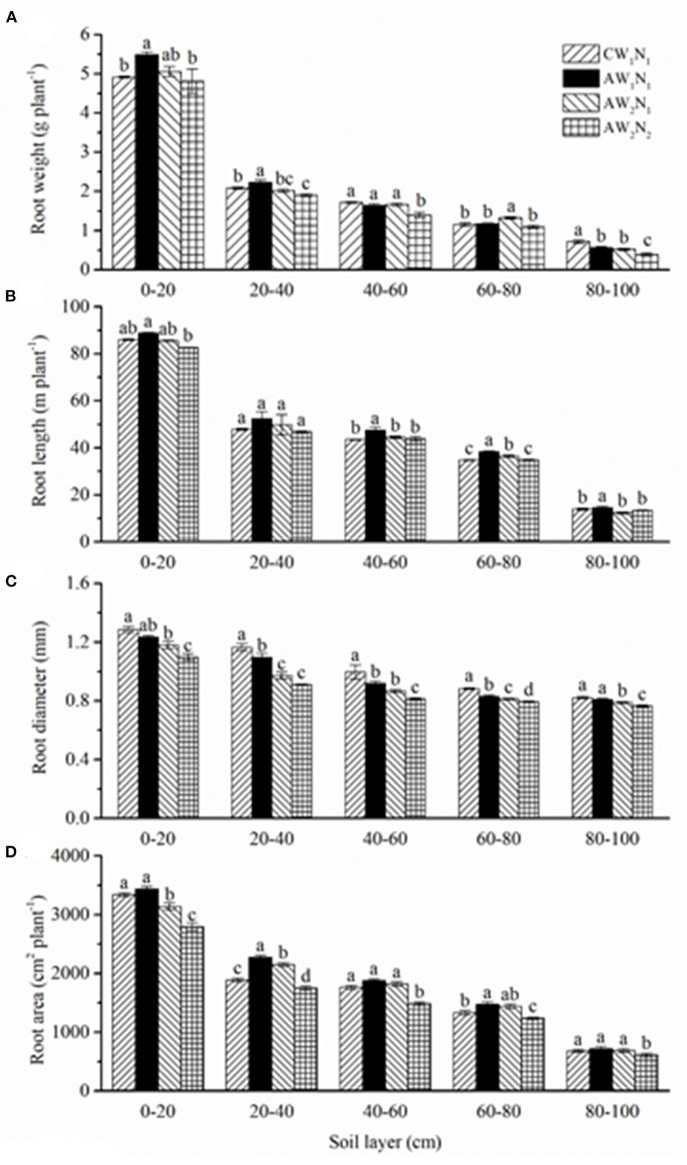
The effects of irrigation and N supply on root growth and distribution in soil layers under alternate partial root-zone drip irrigation. **(A)** Root weight (g plant^−1^), **(B)** root length (m plant^−1^), **(C)** toot diameter (mm) and **(D)** root area (cm^2^ plant^−1^). Values are the means ± SE. Different lowercase letters in the columns denote significant differences among the treatments (*P* ≤ 0.05).

Compared with the CW_1_N_1_ treatment, the root mass in the AW_1_N_1_ treatment increased significantly in the 0–40 cm soil layer, which was similar in the 40–80 cm but decreased in the 80–100 cm soil layer (Figure 3A). The AW_2_N_1_ had a similar root mass in the 0–60 cm soil layer compared with the CW_1_N_1_ treatment. The root mass under APRD in the 80–100 cm soil layer was reduced by 21.4–45.8% compared with that under the CW_1_N_1_ treatment.

Compared with the CW_1_N_1_ treatment, the root length in the AW_1_N_1_ treatment showed no significant difference in the 0–40 cm soil layer but increased by 8.2% significantly in the 40–100 cm (Figure 3B). There was no significant difference in the total root length or root length in different soil layers between AW_2_N_1_ and AW_2_N_2_ except in the 60–80 cm soil layer, where the root length increased significantly in AW_2_N_1_. However, the root length below 40 cm in the soil profile decreased significantly in AW_2_N_1_ and AW_2_N_2_ compared with that of AW_1_N_1_ (Table 2 and Figure 3B).

Compared with CW_1_N_1_, the root diameter tended to decrease under the APRD treatments (Figure 3C), by 6.5% on average in AW_1_N_1_ in the 20–80 cm layer, and 10 and 14.4% in 0–100 cm in AW_2_N_1_ and AW_2_N_2_, respectively.

The variations of the root surface area among the treatments were similar to that of the root length (Figure 3D). Compared with CW_1_N_1_, the root surface area in the soil profile of AW_1_N_1_ tended to increase in the 0–80 cm layer and showed a significant difference in 20–80 cm. Reduction of water and/or N fertilizer under APRD decreased the root surface area, but this decrease became less obvious with the increase in soil depth. Compared with CW_1_N_1_, the root surface area in the AW_2_N_2_ treatment decreased significantly in different soil layers except in 60–80 cm.

### Effects of Irrigation and Nitrogen Supply on Water, Nitrogen, and ^15^N Use Efficiency

At the same fertigation level, AW_1_N_1_ increased the N absorption by 13.8%, and irrigation water use efficiency (IWUE) by 7.1% compared with that of CW_1_N_1_. However, there was no significant difference in the NUE between CW_1_N_1_ and AW_1_N_1_ (Table 3). Under APRD, the total N absorption by the tomato plants decreased with a reduction in irrigation amount and N level, with the highest N absorption observed in AW_1_N_1_. The IWUE of plants treated with AW_2_N_1_ was highest among the treatments. Compared with CW_1_N_1_, AW_2_N_1_ increased the IUWE by 35.6%, but no significant difference in NUE was observed. The AW_2_N_2_ treatment had the highest NUE among all treatments, being 9.4% higher than that of CW_1_N_1_. Similarly, the IWUE was also higher in AW_2_N_2_ than those in CW_1_N_1_ and AW_1_N_1_ treatments.

**Table 3:** The effects of irrigation and N supply on the total plant N absorption, irrigation water (IWUE), and N (NUE) use efficiency under alternate partial root-zone drip N fertigation.

**Treatments**	**Total N (g plant^**−1**^)**	**NUE (g·g^**−1**^)**	**IWUE (kg·m^**−3**^)**	**IWUE (kg·ha^−1^·mm^**−1**^)**
CW_1_N_1_	8.41 ± 0.10b	26.26 ± 0.31bc	83.41 ± 0.56d	834 ± 6d
AW_1_N_1_	9.57 ± 0.11a	27.12 ± 0.31b	89.34 ± 0.47c	893 ± 5c
AW_2_N_1_	8.21 ± 0.23b	25.43 ± 0.32c	113.12 ± 1.44a	1131 ± 14a
AW_2_N_2_	7.06 ± 0.14c	28.74 ± 0.49a	105.53 ± 1.86b	1055 ± 19b

*The values are the means ± SE. The different lowercase letters in the columns denote significant differences among the treatments (P ≤ 0.05)*.

**Figure 4:**
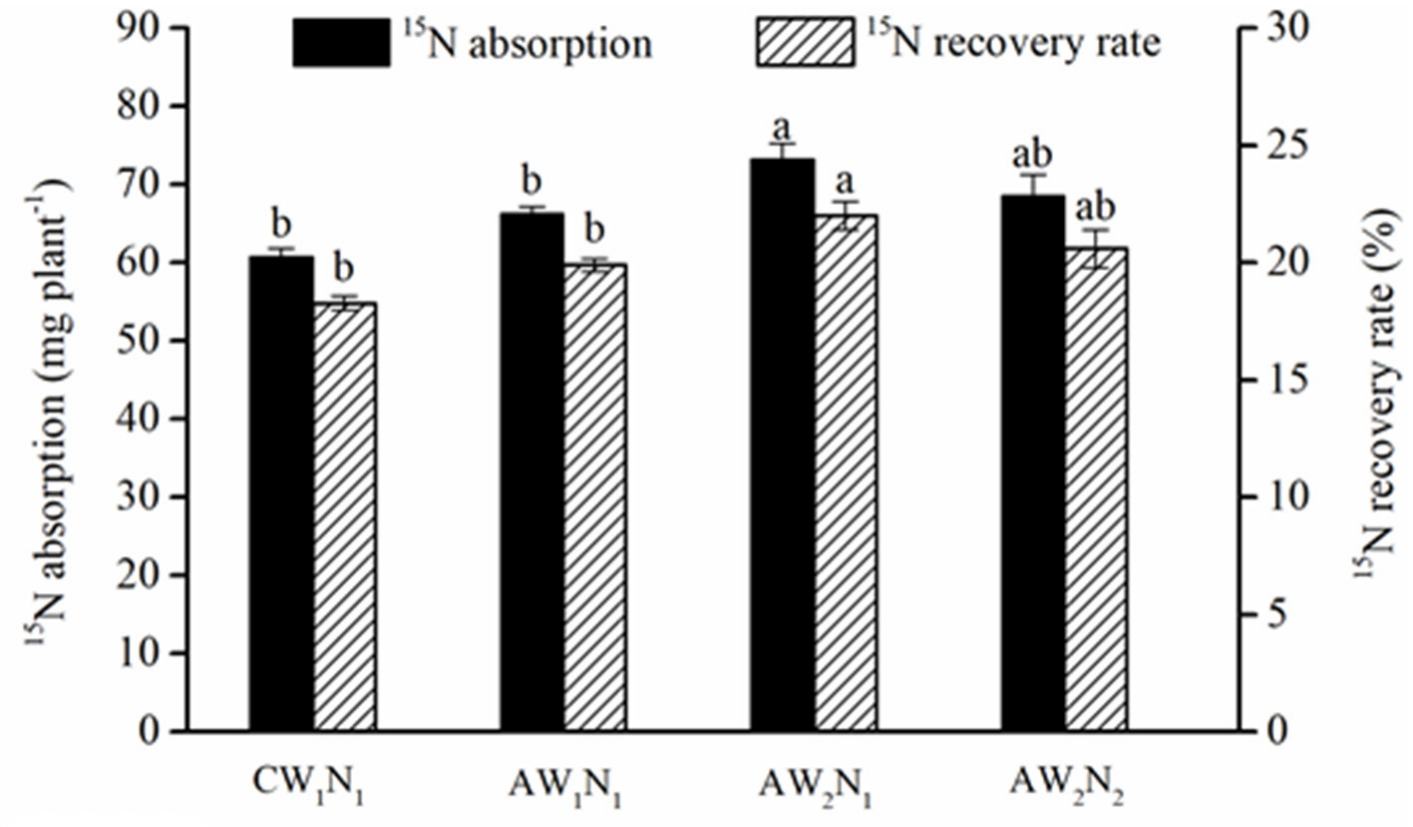
The effects of water and N supply on ^15^N absorption and recovery rate by tomato plants under alternate partial root-zone drip irrigation. Values are the means ± SE. Different lowercase letters in the columns denote significant differences among the treatments (*P* ≤ 0.05).

There was no significant difference in the total ^15^N absorption and ^15^N recovery rate between the treatments CW_1_N_1_ and AW_1_N_1_ (Figure 4). The ^15^N absorption and recovery rate in AW_2_N_1_ was the highest among all the treatments, which increased by 20.5% compared with that of CW_1_N_1_. The total ^15^N absorption and recovery rate showed no significant difference among the treatments of AW_2_N_2_, AW_1_N_1_, and CW_1_N_1_.

### Effects of Irrigation and Nitrogen Supply on ^15^N Distribution in Different Soil Layers

**Figure 5:**
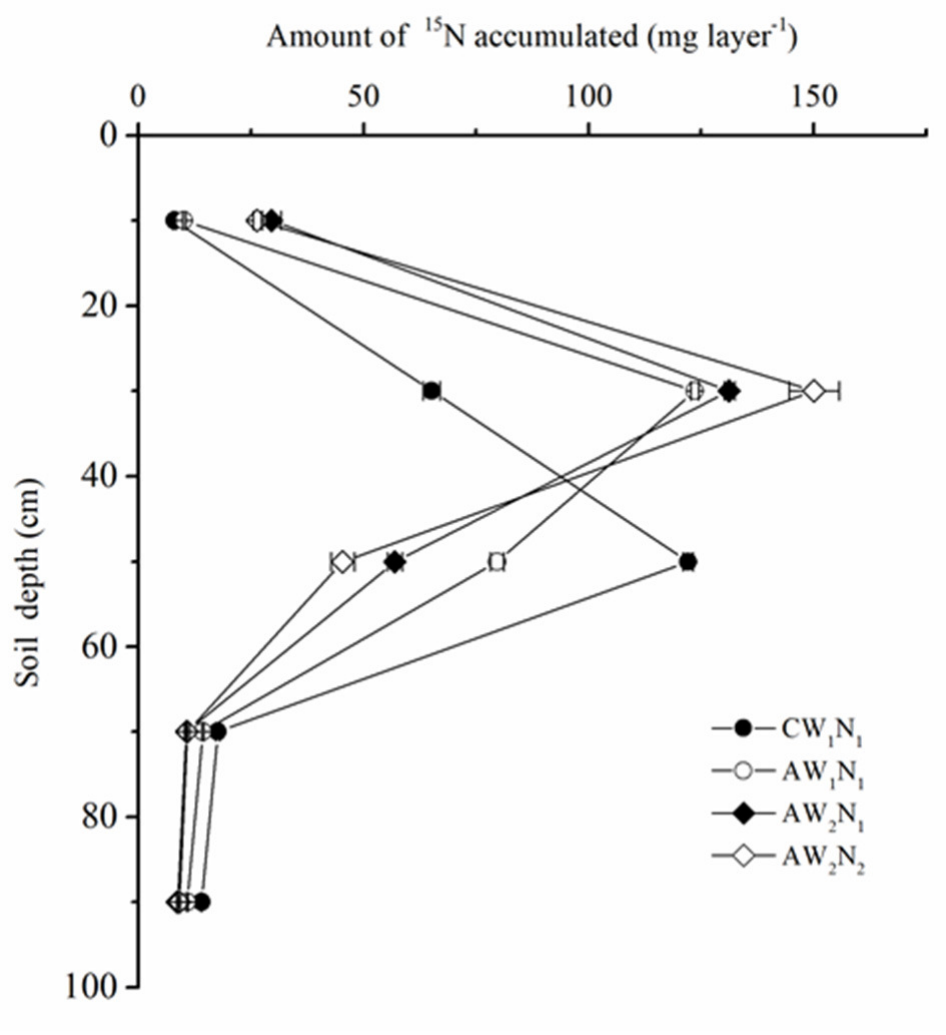
The effects of irrigation and N supply on the distribution of labeled ^15^N in different soil layers under alternate partial root-zone drip irrigation. Values are the means ± SE.

The ^15^N was labeled in the 30–40 cm layer of the soil columns. However, ^15^N was also found in the 0–20 cm layer after harvest (Figure 5), indicating that ^15^N has moved upward and was taken up by plants. In the 0–20 cm soil layer, the ^15^N accumulation in the CW_1_N_1_ treatment was significantly lower than that of the APRD treatment. In the 20–40 cm soil layer, the ^15^N accumulation of CW_1_N_1_ accounted for only 19.3% of the total ^15^N labeled, while AW_1_N_1_, AW_2_N_1_, and AW_2_N_2_ accounted for 36.6, 38.6, and 49.7% of the applied ^15^N, respectively.

The distance between the soil layer where the ^15^N accumulation peak appeared after plant harvest and the layer labeled with K^15^NO_3_ at the beginning of the experiment was considered the movement distance of the residual nitrogen in a specific layer (Wang et al., 2014; Liu et al., 2020). The ^15^N accumulation in the CW_1_N_1_ treatment peaked in the 40–60 cm layer, indicating the downward movement distance of 10–20 cm; while the ^15^N accumulation in all the APRD treatments in 20–40 cm, indicating a slow movement of ^15^N in the soil profile or it moved upward only within 10 cm (as we did not take soil samples every 10 cm per layer) as reported previously (Wang et al., 2014, 2019, 2020). In the layers where the ^15^N accumulation peaked, the ^15^N accumulation in CW_1_N_1_ decreased by 2.1, 8, and 17.7%, respectively, compared with those in AW_1_N_1_, AW_2_N_1_, and AW_2_N_2_ treatments. In APRD with the same nitrogen supply level (N_1_), the ^15^N accumulation increased in the 0–20 and 20–40 cm soil layers, while decreased in the layers below 40 cm in the AW_2_N_1_ treatment as compared with AW_1_N_1_. Under deficient soil water level, the reduction in 50% of the nitrogen supply (AW_2_N_2_) increased the ^15^N accumulation significantly in the 20-40 cm layer, but decreased in the 40–60 cm, with no significant difference observed in ^15^N accumulation in the other layers compared with AW_2_N_1_.

### Effects of Irrigation and Nitrogen Supply on the ^15^N Accumulation and Recovery and Its Loss From the Soil Column

Under the same amount of water and nitrogen supply, the AW_1_N_1_ treatment increased the total ^15^N recovery by 3.7%, while the ^15^N loss rate decreased by 21.3%, and with no significant effects on the ^15^N accumulation in the 0–100 cm soil profile compared with CW_1_N_1_ (Table 4). Under APRD, the reduction in both the water and N fertilizer significantly increased the ^15^N recovery and accumulation in the soil profile, while the ^15^N loss and loss rate decreased by 30%. However, the recovery amount, loss amount, and loss rate were similar between AW_2_N_2_ and AW_2_N_1_.

**Table 4:** The effects of irrigation and N treatments on ^15^N accumulation and recovery and ^15^N loss from the top 0–100 cm soil layer under alternate partial root-zone drip N fertigation.

**Treatments**	**Accumulation amount (mg column^**–1**^)**	**Recovery amount (mg column^**–1**^)**	**Loss amount (mg column^**–1**^)**	**Loss rate (%)**
CW_1_N_1_	226.66 ± 1.60b	287.47 ± 2.46c	50.33 ± 2.46a	14.90 ± 0.73a
AW_1_N_1_	231.85 ± 3.07b	298.21 ± 2.49b	39.59 ± 2.49b	11.72 ± 0.74b
AW_2_N_1_	239.95 ± 2.15a	310.71 ± 1.54a	25.09 ± 1.64c	7.43 ± 0.56c
AW_2_N_2_	241.62 ± 2.14a	310.19 ± 0.72a	27.61 ± 0.92c	8.17 ± 0.22c

*11.9 gram of ^15^N-labeled K^15^NO_3_ with the ^15^N abundance of 20.3% provided 337.8 mg ^15^N to the 30–40 cm soil layer in each column. The total recovery amount of ^15^N was included in the ^15^N accumulated in the 0–100 cm soil and absorbed by the plant. The total loss of ^15^N was calculated from the difference between labeled ^15^N introduced by K^15^NO_3_ and the total recovery amount. The data in the table are the means ± SE. The different lowercase letters in the columns denote significant differences among different treatments (P ≤ 0.05)*.

## Discussion

### Effect of Irrigation and Nitrogen Supply on Plant Growth, WUE, and NUE

In the present study, the crop growth and yield were significantly affected by the water and N application levels and methods. Compared with CW_1_N_1_, APRD significantly increased the plant biomass and fruit mass under the same level of irrigation and N application (AW_1_N_1_) (Table 1). Reducing the irrigation water by one-third decreased the plant biomass significantly, while the fruit yield was not affected in AW_2_N_1_ (Table 1). Therefore, APRD improved the IWUE by 35.6% without any significant yield reduction (Tables 1, 3). This result is in agreement with the advantages of APRD on saving irrigation water reported in several previous studies (Topak et al., 2016; Sezen et al., 2019; Shu et al., 2020). In addition to the improved IUWE, N absorption was also enhanced under APRD (Table 3). Even though the biomass decreased significantly, the total N absorption varied little in AW_2_N_1_ when compared with that in CW_1_N_1_ whereas it decreased by only 16% in AW_2_N_2_, where a 50% reduction in the nitrogen fertilizer occurred (Tables 1, 3).

Several reasons could account for the improved IUWE and N absorption under APRD. Under APRI (including APRD), roots could sense the drying soil, and thus generated root-sourced signals to reduce stomatal opening (Kang and Zhang, 2004; Dodd et al., 2006; Liu et al., 2006). It has been demonstrated that the moderate closure of the stomata significantly inhibited the transpiration rate, but showed little effect on photosynthesis, which can stabilize crop growth and yield and improve WUE under APRI with a substantial reduction in irrigation water (Kang and Zhang, 2004). Moreover, under APRI, the root system in the irrigated side can absorb enough water and nutrients to meet the demands of the plants. The roots under APRD became thinner, which increased the root length and surface area in different layers of the soil profile, especially in the middle and lower layers (Figure 3), which is consistent with our previous reports (Chen et al., 2016; Wang et al., 2019; Liu et al., 2020). Several studies reported that APRI could promote the compensatory and balanced growth of roots in different root zones, stimulate the growth and development of root hairs, and induce more root distribution deeper in the soil [refer to review by Kang and Zhang (2004) and Zhang et al. (2014)]. In addition, the repeated drying/wetting cycles improved the soil aeration, which was conducive to root activity. The increased root growth and root activity could promote the absorption of water and nutrients by plants (Kang and Zhang, 2004; Sarker et al., 2020). Furthermore, APRI could stimulate organic carbon and N mineralization and release more mineral N into the soil solution, which could promote the absorption of N by plants (Sun et al., 2013; Liu et al., 2020). It was reported that APRI (including APRD) could increase the recovery rate of both the fertilizer-N and residual N accumulated in the soil (Wang et al., 2014; Hou et al., 2017; Liu et al., 2020). However, the NUE was not improved in the plants under APRD compared with the conventional irrigation at the same N supply level (AW_1_N_1_, AW_2_N_1_ vs. CW_1_N_1_, Table 3), indicating that APRI-plants absorbed excessive N to ensure growth and yield formation. The NUE increased by 9.4% in AW_2_N_2_ compared with that in CW_1_N_1_ (Table 3). However, it is a general response that the NUE is higher at N_2_ than at N_1_ (Xu et al., 2012), indicating that the increased NUE in AW_2_N_2_ is not an APRD-specific response.

In AW_2_N_1_, the reduced irrigation water decreased the total biomass, but the yield was similar compared with that of the conventional drip irrigation (Table 1). This indicated that the carbon allocation and remobilization from vegetative organs to fruits was enhanced under APRD. Abscisic acid was suggested to play a vital role in the regulation of plant senescence and carbon remobilization (Yang et al., 2000). Higher leaf ABA concentrations were observed throughout the growing season under APRI compared with that under other treatments (Kirda et al., 2004). The enhanced remobilization of photosynthates and higher harvest index induced by APRD were also observed in our previous studies (Zhang et al., 2014; Shu et al., 2020).

### Effect of Irrigation and Nitrogen Supply on Residual Nitrate Loss and Utilization

It was found that when the ^15^N was labeled in the 30–40 cm layer, APRD reduced the ^15^N leaching in the soil column and increased the ^15^N accumulation in the 0–40 cm soil layer by 82.9–141.1% compared with the conventional drip irrigation (Figure 5). Moreover, APRD increased the absorption and utilization of the labeled N significantly under W_2_ coupled with N_2_ application, and the ^15^N loss rate was decreased by 21.3–50.1%, which was the lowest under the deficient irrigation treatments (AW_2_N_1_, AW_2_N_2_) (Table 4). These changes were mainly because under APRI (including in APRD form), the water movement in the soil was different from that of conventional irrigation (Kang and Zhang, 2004; Sarker et al., 2019). The heterogeneous distribution of soil moisture induced by APRI reduced the vertical leakage and promoted the lateral infiltration of soil water. In addition, the irrigation amount has been reduced in most cases under APRI (Kang and Zhang, 2004; Zhang et al., 2014; Wang et al., 2019). All these factors contributed to the reduction in nitrate leaching and N loss under APRI (Wang et al., 2014, 2019). The changes in the ^15^N accumulation peaked soil layer also indicated that the leaching of soil nitrate was weakened by APRI (Figure 5). Root activity and root compensatory growth can be increased by APRI, which can lead to an enhanced nutrient absorption (Chen et al., 2016). In addition to the reduced leaching, APRI or APRD could reduce N loss from the plant-soil system by reducing denitrification and ammonia volatilization (Lei et al., 2009; Han et al., 2014). In the present study, compared with conventional drip irrigation, the total plant biomass, fruit yield, and total N absorption decreased, whereas the absorption and utilization of labeled N increased significantly by decreasing the amount of irrigation water and nitrogen application under APRD (AW_2_N_2_ treatment) (Tables 1, 3 and Figure 4).

Wang et al. (2014, 2019) labeled ^15^N in both the 10–20 and 40–50 cm layers and found that with lowering the ^15^N labeled layer in the soil profile, the absorption and utilization of ^15^N by plants also decreased, while the loss rate of ^15^N from the plant-soil column system increased. The downward leaching distance of ^15^N was shortened under APRI, and even in the case of labeling the ^15^N in the 40–50 cm soil layer, the ^15^N accumulation peaked layer moved upward by 10 cm (Wang et al., 2014; Hou et al., 2017). However, the recovery and loss rate of ^15^N were different between the results reported by Wang et al. (2014, 2019) and Hou et al. (2017), probably due to the differences in the plant growth seasons, ^15^N labeling amount used, and plant growth conditions. In the present study, ^15^N was labeled in the 30–40 cm layer, which was different from the above previous reports, but close to the depth of 40–50 cm as reported by Wang et al. (2014) with a close amount of ^15^N labeled and the same plant growing season. The results showed that under the AW_2_N_1_ treatment, the ^15^N absorption and loss rate from the plant-soil column system was at 22.01 and 7.43% (Figure 4 and Table 4), which was 46.7% higher while 40.8% lower than those of APRI where ^15^N was labeled in the 40–50 cm layer, and even close to those of APRI where ^15^N was labeled in the 10–20 cm layer with the same level of water and N supply as reported by Wang et al. (2014). The distribution of the tomato roots was mainly concentrated at the top 0–20 cm layer (Figure 3) (Wang et al., 2014, 2019; Liu et al., 2020; Shu et al., 2020), thus, the ^15^N labeled in the 10–20 cm layer was more conducive to plant absorption and utilization and to reduce its loss from the plant-soil system (Wang et al., 2014). In the present study, although ^15^N was labeled in the 30-40 cm layer, the ^15^N absorption, utilization, and loss rate in the CW_1_N_1_ were close to those of the conventional irrigation with ^15^N labeled in the 10–20 cm layer as reported by Wang et al. (2014). These results showed that drip irrigation and APRD are more conducive to reducing the leaching and loss of nitrate accumulated in the soil profile, thus promoting plant absorption and its accumulation in soil than the conventional irrigation and APRI (not in the form of APRD here), respectively.

## Conclusions

Compared with CW_1_N_1_, under the same amount of water and N supply, AW_1_N_1_ showed a reduced ^15^N leaching in the soil, promoted root growth, and enhanced the absorption of residual nitrate, thus promoted the tomato growth and yield formation. Compared with CW_1_N_1_, decreasing the irrigation water by 34.1% under APRD (AW_2_N_1_) maintained the total N absorption, tomato yield, and increased the IWUE by 35.6%, the utilization rate of labeled ^15^N by 20.5%, while the loss rate of ^15^N from the plant-soil column was decreased by 50.1%. AW_2_N_2_ increased the absorption of the labeled ^15^N by 12.8%, IWUE by 26.5%, but reduced the yield by 8.8% compared with those under CW_1_N_1_. Even though the N absorption was enhanced, the absorbed-NUE had not improved, indicating that a luxurious absorption of N occurred under APRD compared with the CW_1_N_1_ at the same N supply level. Therefore, it is concluded that APRD can significantly increase IWUE and N absorption, promote its utilization, and thus, reduce the loss of residual N that is accumulated in the soil profile. Thus, APRD is a promising technology for sustainable agriculture production even though the NUE in plants has not been improved.

## Data Availability Statement

The raw data supporting the conclusions of this article will be made available by the authors, without undue reservation.

## Author Contributions

L-ZS and W-JZ conceived and designed the experiments. RL, P-FZ, and J-RZ performed the experiments. RL and ZC analyzed the data and wrote the manuscript. Y-SW designed the experiments and improved the manuscript. All the authors contributed to the article and approved the submitted version.

## Funding

This study was financially supported by the National Natural Science Foundation of China [Grant No. 31572202], the Agricultural Science and Technology Innovation Program [CAAS-ZDRW202002], and the Basic Public Welfare Projects of Zhejiang Province [2017C32082].

## Conflict of Interest

The authors declare that the research was conducted in the absence of any commercial or financial relationships that could be construed as a potential conflict of interest.

## Publisher's Note

All claims expressed in this article are solely those of the authors and do not necessarily represent those of their affiliated organizations, or those of the publisher, the editors and the reviewers. Any product that may be evaluated in this article, or claim that may be made by its manufacturer, is not guaranteed or endorsed by the publisher.
